# The influence of forest habitat type on *Ixodes ricinus* infections with *Rickettsia* spp. in south-western Poland

**DOI:** 10.1016/j.crpvbd.2024.100200

**Published:** 2024-07-08

**Authors:** Dagmara Dyczko, Paweł Błażej, Dorota Kiewra

**Affiliations:** a Department of Microbial Ecology and Acaroentomology, Faculty of Biological Sciences, University of Wroclaw, Przybyszewskiego 63, 51-148, Wroclaw, Poland; b Department of Bioinformatics and Genomics, Faculty of Biotechnology, University of Wroclaw, Joliot-Curie 14a, 50-383, Wroclaw, Poland

**Keywords:** Forest ecosystems, Tick-borne pathogens, Coniferous forest, *Rickettsia helvetica*, *Rickettsia monacensis*

## Abstract

This study investigates the prevalence of *Rickettsia* spp. in *Ixodes ricinus* tick populations in different forest habitat types (broadleaf forest, mixed broadleaf and coniferous forest, and coniferous forest) in south-western Poland. During the survey periods from April to June 2018 and 2019 a total of 494 *I. ricinus* ticks, including 374 nymphs, 60 females and 60 males, were tested for *Rickettsia* infections by nested PCR targeting the *glt*A gene. The overall infection rate was 42.3%; however, we observed statistically significant year-to-year variation. Infection rates varied between tick developmental stages and were significantly influenced by forest habitat type. As assessed by a generalized linear mixed model (GLMM), the highest infection rates were observed in mixed broadleaf and coniferous forests, while coniferous forests had a significant negative effect on infection prevalence. DNA sequencing of selected samples confirmed the predominance of *Rickettsia helvetica* (91.2%) and less frequent *Rickettsia monacensis* (8.8%). This study suggests that the forest habitat types can influence *Rickettsia* spp. infection in tick populations; however, a comprehensive understanding of all factors influencing the level of infection requires future study.

## Highlights

• The overall infection rate of *Rickettsia* spp. in *Ixodes ricinus* was 42.3%.
• Infection rates varied significantly by year and tick developmental stage.
• Forest habitat type influenced *Rickettsia* spp. infection rates in *I. ricinus* nymphs.
• Sequencing confirmed the dominance of *R. helvetica* and lower levels of *R. monacensis.*

## Graphical abstract

**Figure:**
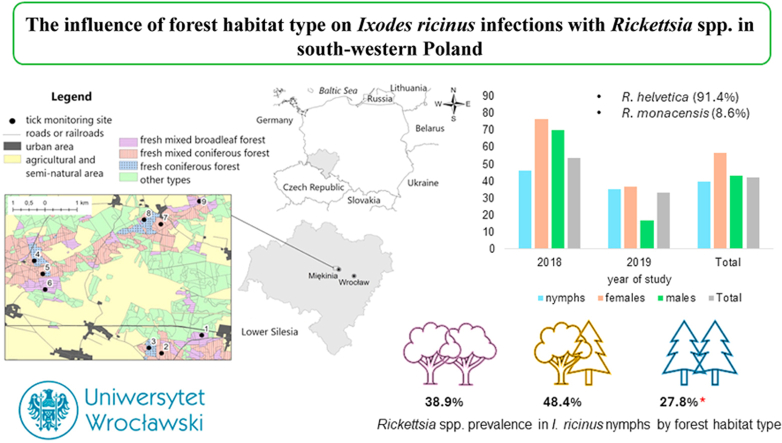

## 1 Introduction

Tick-borne *Rickettsia* spp. of the spotted fever group (SFGR) are responsible for emerging and re-emerging diseases, that pose significant health risks to humans and animals (Rizzoli et al., 2014; Gual-Gonzalez et al., 2024). Understanding the factors that influence the infection rates of ticks with *Rickettsia* spp. is crucial for predicting and managing the tick-borne risk.

In Central Europe, the most important vectors and reservoirs for *Rickettsia* spp. are *Ixodes ricinus*, *Dermacentor reticulatus*, and *D. marginatus* ticks (Karbowiak et al., 2016)*.* They are responsible for both the maintenance and transmission of *Rickettsia* spp., allowing them to survive in natural environments (Ioniță et al., 2016). This dual role is supported by both vertical (transovarial and transstadial) and horizontal (from infected hosts to ticks and *vice versa*) transmission mechanisms, but also by co-feeding (Eremeeva and Dasch, 2015; Fongsaran et al., 2022). Recent studies highlighted the need for a thorough understanding of *Rickettsia*-host-tick interactions, particularly in light of environmental and socio-economic changes that are influencing tick habitats and increasing human cases of tick-borne diseases. This is especially important because *Rickettsia*-host-tick interactions are crucial for both disease transmission and understanding the emergence of new *Rickettsia* species (Portillo et al., 2015; Kim, 2022; Arz et al., 2023).

*Ixodes ricinus* is recognized as one of the most important vector species in the world. It occurs throughout Poland and is the most common tick species parasitizing humans. The geographical distribution of *I. ricinus* is influenced by environmental factors such as temperature and humidity, which affect tick survival and dispersal (Medlock et al., 2013). Previous studies have shown that climate change is likely to expand the suitable habitats for *I. ricinus*, potentially increasing the risk of *Rickettsia* spp. infections and other tick-borne pathogens in new areas (Estrada-Peña et al., 2012; Medlock et al., 2013; Voyiatzaki et al., 2022).

Environmental and habitat conditions play a crucial role in shaping the distribution, density, and infection rates of *I. ricinus*. Forest habitats, which offer both host availability, including reservoir hosts, and favorable microclimatic conditions for tick survival can significantly impact tick-host-pathogen interactions (Ehrmann et al., 2017; Bourdin et al., 2022). The diversity of forest habitats, ranging from broadleaf to coniferous and mixed forests, creates different environmental conditions that can influence tick populations and their pathogen load (Bourdin et al., 2022; Dyczko et al., 2022). For example, canopy cover, humidity, and temperature within these habitats can influence tick activity, survival rates, and host species behavior (Agrillo et al., 2021; Gardiner et al., 2021; Steel et al., 2022). These environmental factors, in turn, affect the likelihood that ticks will acquire *Rickettsia* spp. from the host and subsequently transmit the bacteriae to new hosts (Bourdin et al., 2022; Arz et al., 2023). Broadleaf forests, with their dense undergrowth and moist conditions, provide an ideal environment for ticks and their hosts, leading to higher tick densities and potentially increased rates of pathogen transmission. The abundant leaf litter and soil moisture in these types of forests provide suitable conditions for tick survival and development, while a wide range of mammals and birds serve as hosts for different tick life stages (Gilbert et al., 2012; Tack et al., 2012; Paul et al., 2016; Hofmeester et al., 2017). In contrast, coniferous forests might present a less favorable environment for *I. ricinus* due to lower humidity levels, reduced undergrowth complexity, and fewer suitable hosts, potentially leading to lower tick abundance and a decreased chance of pathogen transmission (Estrada-Peña, 2001; James et al., 2013; Wierzbicka et al., 2016; Dyczko et al., 2022). However, microclimatic conditions, such as temperature and humidity at the forest floor level, can vary significantly within forest types, affecting the survival of ticks and their potential to transmit pathogens, including *Rickettsia* spp. (Boehnke et al., 2017; Gethmann et al., 2020).

Rates of infection of *I. ricinus* with *Rickettsia* spp. are influenced by a variety of factors, including the presence of suitable hosts, environmental conditions, and genetic diversity of both ticks and pathogens (Biernat et al., 2016; Takumi et al., 2019; Numan et al., 2022). In addition, recent studies have shown that *I. ricinus* ticks are not only passive vectors of *Rickettsia* spp. but may have complex interactions with various microorganisms, including symbiotic relationships with bacteria (Guizzo et al., 2023; Wiesinger et al., 2023).

The prevalence of *Rickettsia* spp. in *I. ricinus* ticks also varies significantly between different geographical regions and forest types, suggesting that landscape-level factors play a critical role in disease ecology (Kuo et al., 2015; Hansford et al., 2023). Urbanization and habitat fragmentation, leading to changes in the availability and quality of forest habitats, have been identified as key factors influencing tick populations and the epidemiology of tick-borne diseases (Hansford et al., 2023). Consequently, understanding the specific characteristics of forest habitats that favor or hinder the presence of *Rickettsia*-infected *I. ricinus* ticks is crucial for predicting and managing the risk of tick-borne disease in human and animal populations. In addition, environmental changes such as deforestation, climate change, and human-induced changes in natural habitats can affect the distribution of tick populations and their pathogen load (Medlock et al., 2012; Tack et al., 2013). For example, changes in land use can alter the composition of host species or disrupt the habitats that support the complex life-cycle of *I. ricinus*, thereby affecting the epidemiology of tick-borne diseases (Guo et al., 2019; Diuk-Wasser et al., 2021).

Understanding the relationship between forest type and the prevalence of tick-borne pathogens is crucial for predicting outbreaks of diseases caused by *Rickettsia* spp. and other tick-borne pathogens. Therefore, this study aimed to estimate: (i) the prevalence of infection of *I. ricinus* with *Rickettsia* spp.; and (ii) the influence of forest habitat type on the level of *Rickettsia* spp. infection.

## 2 Materials and methods

### 2.1 Study area

The study was conducted in the Miękinia Forest District of Lower Silesia, SW Poland. The forest habitat type was determined based on land cover maps available in the Forest Data Bank (https://www.bdl.lasy.gov.pl/portal/mapy). Nine sites were included in the study, with three sites in each forest habitat type: sites 1, 6, and 9 in broadleaf forest, sites 2, 5, and 7 in mixed broadleaf and coniferous forest, and sites 3, 4, and 8 in coniferous forest. All designated sites were located within the forest complex to avoid ecotone effects. Detailed site characteristics are described by Dyczko et al. (2022).

### 2.2 Tick collection

Ticks were collected using the standard flagging method during their peak activity in spring, specifically from April to June in both 2018 and 2019. At each of the nine sampling sites, four sample plots of 100 m^2^ were designated. Samples were randomly taken from these designated positions to eliminate time errors, and sampling was carried out on dry and windless days between 9:00 and 15:00 h. The identification of the collected ticks was carried out using a key for species identification under a stereomicroscope (Estrada-Peña et al., 2017). In addition, during tick collection, the temperature and relative humidity were measured 1 m above ground level using a hygrometer (HANNA H19565; Hanna Instruments, Woonsocket, USA).

### 2.3 Identification of *Rickettsia* spp. infections

DNA isolation was carried out using the ammonia method (Rijpkema et al., 1996). Test specimens of ticks were placed individually into Eppendorf tubes and crushed in a 0.7-molar solution of ammonium hydroxide (NH_4_OH). The lysates were stored at −20 °C. A nested PCR method based on the amplification of a fragment of the *glt*A gene was used to detect *Rickettsia* spp. DNA (Prakash et al., 2012). Two sets of primers were used in the reaction. In the first reaction, the primers 877p (5′-GGG GAC -CT GCT CAC GGC GG-3′) and 1258n (5′-ATT GCA AAA AGT ACA GTG AAC C-3′) were used, yielding a product of 381 bp in length. In the second reaction, the primers 896p (5′-GGC TAA TGA AGC AGT GAT AA-3′) and 1233n (5′-GCG ACG GTA TAC CCA TAG C-3′) were used, yielding a product of 338 bp in length. The reaction mixture for a single sample had a volume of 25 μl: 12.5 μl 2× PCR Mix Plus (A&A Biotechnology, Gdynia, Poland), 2.5 μl of each primer (10 μM), 4.5 μl sterile nuclease-free water and 3 μl of template DNA (100 ng) for the first reaction, and 12.5 μl 2× PCR Mix Plus (A&A Biotechnology), 2.5 μl of each primer, 5.5 μl-sterile nuclease-free water and 2 μl of the outer PCR product for nested PCR. In parallel, a positive control was run for each reaction consisting of *Rickettsia* spp. DNA confirmed by sequencing, and nuclease-free water as a negative control. PCR reactions included an initial denaturation at 95 °C for 2 min, 35 cycles each consisting of denaturation at 95 °C for 30 s, primer annealing at 50 °C (primers 877p and 1258n) or 48 °C (primers 896p and 1233n) for 30 s, elongation at 72 °C for 1 min, and a final elongation step at 72 °C for 5 min. The separation of nested PCR products was carried out by electrophoresis on a 1.5% agarose gel with the addition of a nucleic acid stain SimplySafe (Eurx, Gdańsk, Poland) against DNA mass standards (Marker 1: 100–1000 bp; A&A Biotechnology, Gdynia, Poland). The separation of products was carried out at 100 V for 30 min on the Cleaver Scientific CS-300 V omniPAC MIDI Power Supply apparatus. The results of the PCR were viewed under UV light and were archived in computer storage using Quantity One Basic Software (Bio-Rad, Hercules, CA, USA). The presence of a product of 338 bp was considered a positive result.

To check the presence of *Rickettsia* spp., 494 randomly selected ticks of *I. ricinus* (max. of 30 ticks from each site in that year except for sites 4, 7, and 10 of which all collected ticks were tested) including 374 nymphs, 60 females and 60 males were tested, which constituted only a subset of all collected ticks. A total of 34 randomly selected samples that tested positive for nested PCR were subjected to treatment with the EPPiC kit (A&A Biotechnology) and subsequent sequencing (Macrogen, Amsterdam, the Netherlands). The resulting sequences were then compared to existing sequences in GenBank using BLAST. The newly generated sequences were submitted in the GenBank database under the accession numbers PP982421-PP982454.

### 2.4 Phylogenetic analysis

The resulting nucleotide sequences were edited using the DNA Baser Sequence Assembly software (Heracle BioSoft S.R.L., Cluj-Napoca, Romania) and aligned with reference sequences of *Rickettsia* spp. available in GenBank. Phylogenetic analyses were performed using MEGA X software (Kumar et al., 2018). The tree was constructed using maximum likelihood (ML) analysis and bootstrap support was estimated using 1000 replicates.

### 2.5 Statistical analysis

To determine possible relationships between *Rickettsia* spp. infection of ticks and forest habitat type, we decided to assess the data only for nymphs of *I. ricinus* due to an adequate number of specimens. We used GLMM (generalized linear mixed model - negative binomial regression) methodology, which is widely used especially in problems where a response variable is discrete or non-normally distributed in general (Bolker et al., 2009). The statistical analyses were performed in R (R Core Team, 2023).

To assess the relationship between *Rickettsia* spp. infection levels and tick developmental stages (nymphs, females, and males) and between study years, a chi-square test was used with the software Statistica. Statistical significance was set at *P* < 0.05.

## 3 Results

In the period from April and June of 2018 and 2019, a total of 2196 ticks were collected from the nine designated sites. Of these, 2093 were identified as *Ixodes ricinus* (95.3%), 46 as *Dermacentor reticulatus* (2.1%), and 57 as *Haemaphysalis concinna* (2.6%). Detailed information on the results regarding the population structure of the collected ticks, the influence of meteorological factors as well as the influence of the forest habitat type on tick density has already been published by Dyczko et al. (2022).

A total of 494 *I. ricinus* ticks (374 nymphs, 60 females, 60 males) were tested for the presence of *Rickettsia* spp. Adults and nymphs of *I. ricinus* infected with *Rickettsia* spp. were present in all forest habitat types surveyed in both survey seasons. The overall infection rate with *Rickettsia* spp. was 42.3%.

In 2018, a higher overall infection rate was observed (53.6%) compared to 2019 showing a decrease to 33.2% and the differences were statistically significant (*P* < 0.0001). When dissecting the data by developmental stages, nymphs showed a substantial infection rate of 46.3% in 2018 and 35.1% in 2019, with an overall infection rate of 39.8% across both years. Females demonstrated the highest infection rates among the stages, with a decrease from 76.7% in 2018 to 36.7% in 2019, resulting in an overall infection rate of 56.6%. Males exhibited an infection rate of 70% in 2018, which significantly decreased to 16.7% in 2019, resulting in an overall infection rate of 43.3%. Differences in *Rickettsia* spp. infection rates between developmental stages were statistically significant (*P* = 0.049) (Table 1).

**Table 1:** *Rickettsia* spp. infection of *I. ricinus* according to the tick developmental stage, the type of forest habitat, and the year of the study.

Developmental stage	Habitat type	No. infected/No. tested (%)		
		2018	2019	Total
Nymph^a^	BF	43/90 (47.8)	27/90 (30.0)	70/180 (38.9)
	MBCF	26/45 (57.8)	33/77 (42.9)	59/122 (48.4)
	CF	5/25 (20.0)	15/47 (31.9)	20/72 (27.8)
Female		23/30 (76.7)	11/30 (36.7)	34/60 (56.6)
Male		21/30 (70.0)	5/30 (16.7)	26/60 (43.3)
Total		118/220 (53.6)	91/274 (33.2)	209/494 (42.3)

*Abbreviations*: BF, broadleaf forest; MBCF, mixed broadleaf and coniferous forest; CF, coniferous forest.

a The number of ticks tested includes only nymphs.

The observed infection frequency of *I. ricinus* nymphs ranged from 27.8% for coniferous forests to 48.4% for mixed broadleaf and coniferous forests (Table 1). The effect of forest site type on *Rickettsia* spp. infection analyzed using GLMM showed that coniferous forest (coefficient = −0.8899, *P* = 0.00534) had a negative significant effect on the prevalence of *Rickettsia* spp. infection.

**Fig. 1:**
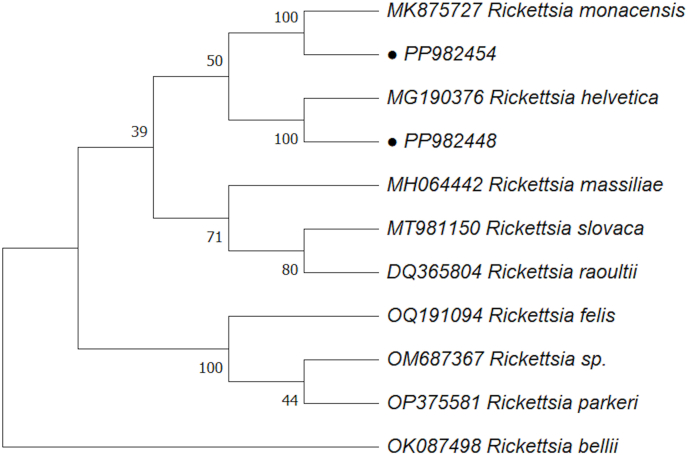
The phylogenetic relationships of *Rickettsia* spp. identified in this study and representatives of different species of the genus *Rickettsia* based on the *glt*A gene and inferred by maximum likelihood. Numbers next to branches indicate bootstrap values based on 1000 replicates. Sequences obtained in this study are marked with a circle.

Sequencing of randomly selected samples (34/209) confirmed the dominant presence of *R. helvetica* (31/34, 91.2%) and less frequent *R. monacensis* (3/34, 8.8%), including the partial sequences of the *glt*A gene were 100% (313/313 nt) identical with a partial sequence of *R. monacensis* (GenBank: MK875727.1) for one sample and 100% (313/313 nt) identical with partial sequences of *R. helvetica* (GenBank: MG190376.1) for another sample (Fig. 1).

## 4 Discussion

The influence of forest habitat types on the prevalence and transmission dynamics of *Rickettsia* spp. infections represents a critical area of research in the field of zoonotic diseases. Forest ecosystems, characterized by their biodiversity and diverse ecological characteristics, play an important role in shaping the interactions between vectors, hosts, and pathogens (Halos et al., 2010; Ogrzewalska et al., 2011).

The present study provides important insights into the spatial and temporal dynamics of *Rickettsia* spp. infection in *Ixodes ricinus* tick populations in different forest habitats in southwestern Poland, indicating a significant impact of forest type on infection level. Based on a two-year study we found that *I. ricinus* nymphs collected in coniferous forests seem to be less infected with *Rickettsia* spp. compared with nymphs harvested in broadleaf and mixed broadleaf and coniferous forests. However, at the same time, it is worth emphasizing that the level of tick infection varies significantly between years, which indicates the complexity of factors influencing the level of infection. In Europe, the prevalence of *Rickettsia* spp. in ticks is highly variable and site-dependent, with infection rates ranging from as low as 0.5% to as high as 66% (Oechslin et al., 2017). Our results highlight a remarkable prevalence of *Rickettsia* spp. with an overall infection rate of 42.3% among the collected tick samples. This finding is consistent with previous studies showing the widespread distribution of *Rickettsia* spp. in tick populations across Europe (Oteo and Portillo, 2012; Balážová et al., 2022; Ivan et al., 2022; Arz et al., 2023). Similar studies conducted in different parts of Europe have consistently shown a variable prevalence of *Rickettsia* spp. in *I. ricinus* ticks, highlighting the importance of habitat type in determining tick infection rates (Coipan et al., 2013; Welc-Falęciak et al., 2014; Szekeres et al., 2015; Biernat et al., 2016; Scarpulla et al., 2018; Stańczak et al., 2018; Krawczyk et al., 2022; Arz et al., 2023). Furthermore, our study is consistent with the findings of Špitalská et al. (2014) and Zając et al. (2023), who observed *Rickettsia*-infected *I. ricinus* ticks in both urban and natural forest areas, indicating that these pathogens are widely distributed in different ecosystems.

The variable infection rates over the two years, with a higher rate in 2018 (53.6%) compared to 2019 (33.2%), suggest potential inter-annual variations in environmental conditions or host availability that could influence *Rickettsia* spp. prevalence. Previous research has shown that changes in temperature, humidity, rainfall, and other climatic factors can directly affect the life-cycle and distribution of ticks, thereby altering patterns of *Rickettsia* transmission, with warmer and wetter climates generally favoring higher tick populations (Alkishe and Peterson, 2022; Cunze et al., 2022; Dyczko et al., 2022; Domatskiy and Sivkova, 2023). These conditions can lead to an increase in the incidence of tick-borne diseases, including those caused by *Rickettsia* spp. On the other hand, extreme weather events (heat, cold, and flooding), possibly linked to climate change, may disrupt tick populations and their activity patterns, potentially reducing the prevalence of *Rickettsia* in certain areas (Gray et al., 2009; Ogden et al., 2021; Deng et al., 2022). The availability of suitable hosts is known to influence the maintenance and spread of *Rickettsia* within ecosystems, as supported by previous studies (Satjanadumrong et al., 2019; Gibb et al., 2020; Kim, 2022). Although this study does not directly assess the impact of host availability, understanding various ecological factors influencing the prevalence of *Rickettsia* spp. in ticks may contribute to understanding complex relationships. For instance, changes in land cover and agricultural practices may affect host availability and, consequently, the transmission cycle of *Rickettsia* spp. (Gilbert, 2021; Rocha et al., 2022). Future research could investigate these factors to further elucidate their roles in *Rickettsia* spp. transmission.

Our study covered only two sampling years and showed year-to-year variation in *Rickettsia* prevalence. However, it is essential to note that other studies, such as the study by Coipan et al. (2013) over a period of ten years and Klitgaard et al. (2019) over two years, have found no significant temporal variation for *Rickettsia*. This discrepancy in findings suggests that the dynamics of *Rickettsia* spp. transmission may vary significantly between different geographical regions. Consequently, this variation could lead to distinct risk management approaches depending on the country. Therefore, understanding these regional differences is crucial for developing effective public health strategies. In addition, understanding the variability in infection rates in different populations may help to better understand the ecological dynamics of vector-borne diseases and how these processes are affected by environmental change.

Our research indicates a significant impact of the developmental stage on the level of infection. Adults, especially females, appeared to be more infected than the nymphs (56.6%, 43.3% and 39.8%, respectively). Higher infection rates in females and males may be influenced by their longer lifespan and greater number of blood meals compared to nymphs, which increases their exposure to infected hosts (Gray et al., 2009). This is in line with research conducted previously in southwestern France (Akl et al., 2019) and northern and central Germany (Schicht et al., 2012; Arz et al., 2023), where it was found that adult ticks are more infected than nymphs. However, other studies have not found such differences or have only found them in certain years. This may be due to the vertical transmission of *R. helvetica* and its establishment in the tick vector in these areas (Sprong et al., 2009; Severinsson et al., 2010; Venclikova et al., 2014; Klitgaard et al., 2019). The lower infection rate of *I. ricinus* nymphs with *Rickettsia* spp. estimated in our study in coniferous forests compared to broadleaf and mixed broadleaf and coniferous forests, could be related to microclimatic conditions or differences in host species composition. These factors also affect the abundance of collected ticks in coniferous forests as shown by Dyczko et al. (2022). The influence of both season and habitat on tick infection rate with *Rickettsia* spp. is also indicated by other researchers. A study conducted in Denmark found higher infection rates in May and in ecotone areas compared to other months and habitats such as spruce or beech forests (Kantsø et al., 2010). In contrast, other studies in Europe found no statistical effect of habitat type on *Rickettsia* spp. prevalence in *I. ricinus* ticks (Špitalská et al., 2014; Knoll et al., 2021; Bourdin et al., 2022; Arz et al., 2023). A lack of significant relationship between habitat type and the presence of *Rickettsia* in ticks collected from rodents was shown in southwest Tennessee, USA, suggesting that habitat type may not be a critical factor in the prevalence of *Rickettsia* spp. in ticks in this region (Butler et al., 2022); however, the availability of reservoir hosts may play an important role. The negative significant effect of coniferous forests on *Rickettsia* spp. prevalence suggests that these environments may be less conducive to the survival or spread of reservoirs of *Rickettsia* spp. or vectors. This aspect warrants further investigation, focusing on environmental and ecological factors that may limit or facilitate the presence of *Rickettsia* spp. in specific forest types.

The predominance of *R. helvetica* (91.2%) in the ticks, confirmed by sequencing, is consistent with previous studies indicating that this species is a common pathogen in European *I. ricinus* tick populations (Rizzoli et al., 2014; Welc-Falęciak et al., 2014; Stańczak et al., 2018; Knoll et al., 2021). *Rickettsia helvetica* belongs to the species of the spotted fever group (SFG), mainly transmitted by *Ixodes* ticks (Scarpulla et al., 2018). Mice, deer, and wild boar may play an important role in the transmission of *R. helvetica*; these may act as reservoir hosts and be involved in the further geographical spread of the pathogen (Sprong et al., 2009; Arz et al., 2023). However, the reservoir hosts of specific *Rickettsia* spp. are still not well defined, and further research is needed to clarify the full importance of potential host species in maintaining infection in the environment. The presence of *R. monacensis* (8.8%), although less frequent, indicates a complex ecological network of vector-host-pathogen interactions and adds to the understanding of the diversity of *Rickettsia* spp. in tick populations in Poland, highlighting the need for continued surveillance and research to understand the distribution patterns and public health implications of these pathogens (Parola et al., 2013; Rymaszewska and Piotrowski, 2013; Portillo et al., 2015; Biernat et al., 2016).

The results of this study are crucial for public health considerations, as *Rickettsia* spp. can cause serious human diseases. The consistent detection of *R. helvetica* across different studies and regions highlights its prevalence and potential impact on public health (Kantsø et al., 2010; Eremeeva and Dasch, 2015; Scarpulla et al., 2018; Maître et al., 2022). Understanding the factors that influence the prevalence and distribution of *Rickettsia* spp. can help formulate strategies for monitoring and controlling tick-borne rickettsial diseases.

## 5 Conclusions

The present study suggests a possible spatial and temporal variability in *Rickettsia* spp. infection rates within *I. ricinus* tick populations. These findings contribute to a broader understanding of the epidemiology of tick-borne diseases. Future research should continue exploring the complex interplay between tick biology, host-pathogen dynamics, and environmental factors to better predict and mitigate the public health impact of these pathogens.

## Funding

This research did not receive any specific grant from funding agencies in the public, commercial, or not-for-profit sectors.

## Ethical approval

Not applicable.

## CRediT authorship contribution statement

**Dagmara Dyczko:** Conceptualization, Methodology, Investigation, Data curation, Writing – original draft, Writing – review & editing. **Paweł Błażej:** Formal analysis, Writing – review & editing. **Dorota Kiewra:** Conceptualization, Writing – review & editing.

## Declaration of competing interests

The authors declare that they have no known competing financial interests or personal relationships that could have appeared to influence the work reported in this paper.

## Data Availability

The data supporting the conclusions of this article are included within the article. The newly generated sequences were submitted to the GenBank database under the accession numbers PP982421-PP982454.

## References

[bib1] Agrillo E., Filipponi F., Pezzarossa A., Casella L., Smiraglia D., Orasi A. (2021). Earth observation and biodiversity big data for forest habitat types classification and mapping. Rem. Sens..

[bib2] Akl T., Bourgoin G., Souq M.L., Appolinaire J., Poirel M.T., Gibert P. (2019). Detection of tick-borne pathogens in questing *Ixodes ricinus* in the French Pyrenees and first identification of *Rickettsia monacensis* in France. Parasite.

[bib3] Alkishe A., Peterson A.T. (2022). Climate change influences on the geographic distributional potential of the spotted fever vectors *Amblyomma maculatum* and *Dermacentor andersoni*. PeerJ.

[bib4] Arz C., Król N., Imholt C., Jeske K., Rentería-Solís Z., Ulrich R.G. (2023). Spotted fever group rickettsiae in ticks and small mammals from grassland and forest habitats in central Germany. Pathogens.

[bib5] Balážová A., Földvári G., Bilbija B., Nosková E., Široký P. (2022). High prevalence and low diversity of *Rickettsia* in *Dermacentor reticulatus* ticks, Central Europe. Emerg. Infect. Dis..

[bib6] Biernat B., Stańczak J., Michalik J., Sikora B., Wierzbicka A. (2016). Prevalence of infection with *Rickettsia helvetica* in *Ixodes ricinus* ticks feeding on non-rickettsiemic rodent hosts in sylvatic habitats of west-central Poland. Ticks Tick Borne Dis..

[bib7] Boehnke D., Gebhardt R., Petney T., Norra S. (2017). On the complexity of measuring forests microclimate and interpreting its relevance in habitat ecology: The example of *Ixodes ricinus* ticks. Parasites Vectors.

[bib8] Bolker B.M., Brooks M.F., Clark C.J., Geange S.W., Poulsen J.R., Stevens M.H., White J.S. (2009). Generalized linear mixed models: A practical guide for ecology and evolution. Trends Ecol. Evol..

[bib9] Bourdin A., Bord S., Durand J., Galon C., Moutailler S., Scherer-Lorenzen M., Jactel H. (2022). Forest diversity reduces the prevalence of pathogens transmitted by the tick *Ixodes ricinus*. Front. Ecol. Evol..

[bib10] Butler R.A., Trout Fryxell R.T., Kennedy M.L., Houston A.E., Bowers E.K., Coons L.B. (2022). No relationship observed between habitat type and *Rickettsia* presence in ectoparasites collected from rodents in southwestern Tennessee. SW. Nat..

[bib11] Coipan E.C., Jahfari S., Fonville M., Maassen C.B., van der Giessen J., Takken W. (2013). Spatiotemporal dynamics of emerging pathogens in questing *Ixodes ricinus*. Front. Cell. Infect. Microbiol..

[bib12] Cunze S., Glock G., Kochmann J., Klimpel S. (2022). Ticks on the move - climate change-induced range shifts of three tick species in Europe: Current and future habitat suitability for *Ixodes ricinus* in comparison with *Dermacentor reticulatus* and *Dermacentor marginatus*. Parasitol. Res..

[bib13] Deng B., Rui J., Liang S.Y., Li Z.F., Li K., Lin S. (2022). Meteorological factors and tick density affect the dynamics of SFTS in Jiangsu Province, China. PLoS Negl. Trop. Dis..

[bib14] Diuk-Wasser M.A., VanAcker M.C., Fernandez M.P. (2021). Impact of land use changes and habitat fragmentation on the eco-epidemiology of tick-borne diseases. J. Med. Entomol..

[bib15] Domatskiy V.N., Sivkova E.I. (2023). The influence of climatogeographic conditions on the expansion of the range of *Ixodes* ticks. Entomol. Appl. Sci. Lett..

[bib16] Dyczko D., Kiewra D., Kolanek A., Błażej P. (2022). The influence of local environmental factors in southwestern Poland on the abundance of *Ixodes ricinus* and prevalence of infection with *Borrelia burgdorferi s.l.* and *B. miyamotoi*. Parasitol. Res..

[bib17] Ehrmann S., Liira J., Gärtner S., Hansen K., Brunet J., Cousins S. (2017). Environmental drivers of *Ixodes ricinus* abundance in forest fragments of rural European landscapes. BMC Ecol..

[bib18] Eremeeva M.E., Dasch G.A. (2015). Challenges posed by tick-borne *Rickettsia*e: Eco-epidemiology and public health implications. Front. Public Health.

[bib19] Estrada-Peña A. (2001). Distribution, abundance, and habitat preferences of *Ixodes ricinus* (Acari: Ixodidae) in northern Spain. J. Med. Entomol..

[bib20] Estrada-Peña A., Ayllón N., de la Fuente J. (2012). Impact of climate trends on tick-borne pathogen transmission. Front. Physiol..

[bib21] Estrada-Peña A., Mihalca A.D., Petney N.T. (2017).

[bib22] Fongsaran C., Jirakanwisal K., Tongluan N., Latour A., Healy S., Christofferson R.C., Macaluso K.R. (2022). The role of cofeeding arthropods in the transmission of *Rickettsia felis*. PLoS Negl. Trop. Dis..

[bib23] Gardiner M., Perry K.I., Riley C.B., Turo K.J., Delgado de la Flor Y.A., Sivakoff F.S. (2021). Community science data suggests that urbanization and forest habitat loss threaten aphidophagous native lady beetles. Ecol. Evol..

[bib24] Gethmann J., Hoffmann B., Kasbohm E., Süss J., Habedank B., Conraths F.J. (2020). Research paper on abiotic factors and their influence on *Ixodes ricinus* activity - observations over a two-year period at several tick collection sites in Germany. Parasitol. Res..

[bib25] Gibb R., Redding D.W., Chin K.Q., Donnelly C.A., Blackburn T.M., Newbold T., Jones K.E. (2020). Zoonotic host diversity increases in human-dominated ecosystems. Nature.

[bib26] Gilbert L. (2021). The impacts of climate change on ticks and tick-borne disease risk. Annu. Rev. Entomol..

[bib27] Gilbert L., Maffey G.L., Ramsay S.L., Hester A.J. (2012). The effect of deer management on the abundance of *Ixodes ricinus* in Scotland. Ecol. Appl..

[bib28] Gray J.S., Dautel H., Estrada-Peña A., Kahl O., Lindgren E. (2009). Effects of climate change on ticks and tick-borne diseases in Europe. Interdiscip. Perspect. Infect. Dis..

[bib29] Gual-Gonzalez L., Torres M.E., Self S.C.W., Cantillo-Barraza O., Nolan M.S. (2024). Spotted fever group *Rickettsia* spp. molecular and serological evidence among Colombian vectors and animal hosts: A historical review. Insects.

[bib30] Guizzo M., Hatalová T., Frantová H., Zurek L., Kopáček P., Perner J. (2023). *Ixodes ricinus* ticks have a functional association with *Midichloria mitochondrii*. Front. Cell. Infect. Microbiol..

[bib31] Guo F., Bonebrake T.C., Gibson L. (2019). Land-use change alters host and vector communities and may elevate disease risk. EcoHealth.

[bib32] Halos L., Bord S., Cotté V., Gasqui P., Abrial D., Barnouin J. (2010). Ecological factors characterizing the prevalence of bacterial tick-borne pathogens in *Ixodes ricinus* ticks in pastures and woodlands. Appl. Environ. Microbiol..

[bib33] Hansford K.M., Gillingham E.L., Vaux A.G.C., Cull B., McGinley L., Catton M. (2023). Impact of green space connectivity on urban tick presence, density, and *Borrelia*-infected ticks in different habitats and seasons in three cities in southern England. Ticks Tick Borne Dis..

[bib34] Hofmeester T.R., Sprong H., Jansen P.A., Prins H.H.T., van Wieren S.E. (2017). Deer presence rather than abundance determines the population density of the sheep tick, *Ixodes ricinus*, in Dutch forests. Parasites Vectors.

[bib81] Ioniță M., Silaghi C., Mitrea I., Edouard S., Parola P., Pfister K. (2016). Molecular detection of *Rickettsia**conorii* and other zoonotic spotted fever group rickettsiae in ticks, Romania. Ticks Tick Borne Dis.

[bib36] Ivan T., Matei I.A., Novac C.Ș., Kalmár Z., Borșan S.D., Panait L.C. (2022). Spotted fever group *Rickettsia* spp. diversity in ticks and the first report of *Rickettsia hoogstraalii* in Romania. Vet. Sci..

[bib37] James M.C., Bowman A.S., Forbes K.J., Lewis F., McLeod J.E., Gilbert L. (2013). Environmental determinants of *Ixodes ricinus* ticks and the incidence of *Borrelia burgdorferi sensu lato*, the agent of Lyme borreliosis, in Scotland. Parasitology.

[bib38] Kantsø B., Svendsen C., Jensen P., Vennestrøm J., Krogfelt K.A. (2010). Seasonal and habitat variation in the prevalence of *Rickettsia helvetica* in *Ixodes ricinus* ticks from Denmark. Ticks Tick Borne Dis..

[bib39] Karbowiak G., Biernat B., Stańczak J., Szewczyk T., Werszko J. (2016). The role of particular tick developmental stages in the circulation of tick-borne pathogens affecting humans in Central Europe. 3. Rickettsiae. Ann. Parasitol..

[bib40] Kim H.K. (2022). *Rickettsia*-host-tick interactions: Knowledge advances and gaps. Infect. Immun..

[bib41] Klitgaard K., Kjær L.J., Isbrand A., Hansen M.F., Bødker R. (2019). Multiple infections in questing nymphs and adult female *Ixodes ricinus* ticks collected in a recreational forest in Denmark. Ticks Tick Borne Dis..

[bib42] Knoll S., Springer A., Hauck D., Schunack B., Pachnicke S., Strube C. (2021). Regional, seasonal, biennial, and landscape-associated distribution of *Anaplasma phagocytophilum* and *Rickettsia* spp. infections in *Ixodes* ticks in northern Germany and implications for risk assessment at larger spatial scales. Ticks Tick Borne Dis..

[bib43] Krawczyk A.I., Röttjers L., Fonville M., Takumi K., Takken W., Faust K., Sprong H. (2022). Quantitative microbial population study reveals geographical differences in bacterial symbionts of *Ixodes ricinus*. Microbiome.

[bib44] Kumar S., Stecher G., Li M., Knyaz C., Tamura K. (2018). Mega X: Molecular Evolutionary Genetics Analysis across computing platforms. Mol. Biol. Evol..

[bib45] Kuo C.-C., Shu P.-Y., Mu J., Lee P.-L., Wu Y.-W., Chung C.-K., Wang H. (2015). Widespread *Rickettsia* spp. infections in ticks (Acari: Ixodoidea) in Taiwan. J. Med. Entomol..

[bib46] Maître A., Wu-Chuang A., Mateos-Hernández L., Foucault-Simonin A., Moutailler S., Paoli J.C. (2022). *Rickettsia helvetica* infection is associated with microbiome modulation in *Ixodes ricinus* collected from humans in Serbia. Sci. Rep..

[bib47] Medlock J., Hansford K., Bormane A., Derdáková M., Estrada-Peña A., George J.-C. (2013). Driving forces for changes in geographical distribution of *Ixodes ricinus* ticks in Europe. Parasites Vectors.

[bib48] Medlock J.M., Shuttleworth H., Copley V., Hansford K.M., Leach S. (2012). Woodland biodiversity management as a tool for reducing human exposure to *Ixodes ricinus* ticks: A preliminary study in an English woodland. J. Vector Ecol..

[bib49] Numan M., Islam N., Adnan M., Safi S.Z., Chitimia-Dobler L., Labruna M., Ali A. (2022). First genetic report of *Ixodes kashmiricus* and associated *Rickettsia* sp. Parasites Vectors.

[bib50] Oechslin C.P., Heutschi D., Lenz N., Tischhauser W., Péter O., Rais O. (2017). Prevalence of tick-borne pathogens in questing *Ixodes ricinus* ticks in urban and suburban areas of Switzerland. Parasites Vectors.

[bib51] Ogden N.H., Beard C.B., Ginsberg H.S., Tsao J.I. (2021). Possible effects of climate change on ixodid ticks and the pathogens they transmit: Predictions and observations. J. Med. Entomol..

[bib52] Ogrzewalska M., Uezu A., Jenkins C., Labruna M. (2011). Effect of forest fragmentation on tick infestations of birds and tick infection rates by *Rickettsia* in the Atlantic Forest of Brazil. EcoHealth.

[bib53] Oteo J., Portillo A. (2012). Tick-borne rickettsioses in Europe. Ticks Tick Borne Dis..

[bib54] Parola P., Paddock C.D., Socolovschi C. (2013). Update on tick-borne rickettsioses around the world: A geographic approach. Clin. Microbiol. Rev..

[bib55] Paul R.E., Cote M., Le Naour E., Bonnet S.I. (2016). Environmental factors influencing tick densities over seven years in a French suburban forest. Parasites Vectors.

[bib56] Portillo A., Santibáñez S., García-Álvarez L., Palomar A., Oteo J. (2015). Rickettsioses in Europe. Microb. Infect..

[bib57] Prakash J.A., Sohan Lal T., Rosemol V., Verghese V.P., Pulimood S.A., Reller M. (2012). Molecular detection and analysis of spotted fever group *Rickettsia* in patients with fever and rash at a tertiary care centre in Tamil Nadu, India. Pathog. Glob. Health.

[bib58] R Core Team (2023). https://www.R-project.org/.

[bib59] Rijpkema S., Golubić D., Molkenboer M., Verbeek-De Kruif N., Schellekens J. (1996). Identification of four genomic groups of *Borrelia burgdorferi sensu lato* in *Ixodes ricinus* ticks collected in a Lyme borreliosis endemic region of northern Croatia. Exp. Appl. Acarol..

[bib60] Rizzoli A., Silaghi C., Obiegala A., Rudolf I., Hubálek Z., Földvári G. (2014). *Ixodes ricinus* and its transmitted pathogens in urban and peri-urban areas in Europe: New hazards and relevance for public health. Front. Public Health.

[bib61] Rocha S.C., Velásquez C.V., Aquib A., Al-Nazal A., Parveen N. (2022). Transmission cycle of tick-borne infections and co-infections, animal models and diseases. Pathogens.

[bib62] Rymaszewska A., Piotrowski M. (2013). Use of DNA sequences for *Rickettsia* identification in *Ixodes ricinus* ticks: The first detection of *Rickettsia monacensis* in Poland. Microb. Infect..

[bib63] Satjanadumrong J., Robinson M.T., Hughes T., Blacksell S.D. (2019). Distribution and ecological drivers of spotted fever group *Rickettsia* in Asia. EcoHealth.

[bib64] Scarpulla M., Barlozzari G., Salvato L., De Liberato C., Lorenzetti R., Macrì G. (2018). *Rickettsia helvetica* in human-parasitizing and free-living *Ixodes ricinus* from urban and wild green areas in the metropolitan city of Rome, Italy. Vector Borne Zoon. Dis..

[bib65] Schicht S., Schnieder T., Strube C. (2012). *Rickettsia* spp. and coinfections with other pathogenic microorganisms in hard ticks from northern Germany. J. Med. Entomol..

[bib66] Severinsson K., Jaenson T.G., Pettersson J., Falk K., Nilsson K. (2010). Detection and prevalence of *Anaplasma phagocytophilum* and *Rickettsia helvetica* in *Ixodes ricinus* ticks in seven study areas in Sweden. Parasites Vectors.

[bib67] Špitalská E., Boldiš V., Derdáková M., Selyemová D., Rusňáková Tarageľová V. (2014). Rickettsial infection in *Ixodes ricinus* ticks in urban and natural habitats of Slovakia. Ticks Tick Borne Dis..

[bib68] Sprong H., Wielinga P.R., Fonville M., Reusken C., Brandenburg A.H., Borgsteede F. (2009). *Ixodes ricinus* ticks are reservoir hosts for *Rickettsia helvetica* and potentially carry flea-borne *Rickettsia* species. Parasites Vectors.

[bib69] Stańczak J., Biernat B., Racewicz M., Zalewska M., Matyjasek A. (2018). Prevalence of different *Rickettsia* spp. in *Ixodes ricinus* and *Dermacentor reticulatus* ticks (Acari: Ixodidae) in north-eastern Poland. Ticks Tick Borne Dis..

[bib70] Steel Z., Jones G.M., Collins B., Green R., Koltunov A., Purcell K. (2022). Mega-disturbances cause rapid decline of mature conifer forest habitat in California. Ecol. Appl..

[bib71] Szekeres S., van Leeuwen A., Rigó K., Jablonszky M., Majoros G., Sprong H., Földvári G. (2015). Prevalence and diversity of human pathogenic *Rickettsia*e in urban *versus* rural habitats, Hungary. Exp. Appl. Acarol..

[bib72] Tack W., Madder M., Baeten L., De Frenne P., Verheyen K. (2012). The abundance of *Ixodes ricinus* ticks depends on tree species composition and shrub cover. Parasitology.

[bib73] Tack W., Madder M., Baeten L., Vanhellemont M., Verheyen K. (2013). Shrub clearing adversely affects the abundance of *Ixodes ricinus* ticks. Exp. Appl. Acarol..

[bib74] Takumi K., Sprong H., Hofmeester T.R. (2019). Impact of vertebrate communities on *Ixodes ricinus*-borne disease risk in forest areas. Parasites Vectors.

[bib75] Venclikova K., Rudolf I., Mendel J., Betasova L., Hubalek Z. (2014). Rickettsiae in questing *Ixodes ricinus* ticks in the Czech Republic. Ticks Tick Borne Dis..

[bib76] Voyiatzaki C., Papailia S.I., Venetikou M.S., Pouris J., Tsoumani M.E., Papageorgiou E.G. (2022). Climate changes exacerbate the spread of *Ixodes ricinus* and the occurrence of Lyme borreliosis and tick-borne encephalitis in Europe - how climate models are used as a risk assessment approach for tick-borne diseases. Int. J. Environ. Res. Publ. Health.

[bib77] Welc-Falęciak R., Kowalec M., Karbowiak G., Bajer A., Behnke J., Siński E. (2014). *Rickettsiaceae* and *Anaplasmataceae* infections in *Ixodes ricinus* ticks from urban and natural forested areas of Poland. Parasites Vectors.

[bib78] Wierzbicka A., Rączka G., Skorupski M., Michalik J., Lane R. (2016). Human behaviors elevating the risk of exposure to *Ixodes ricinus* larvae and nymphs in two types of lowland coniferous forests in west-central Poland. Ticks Tick Borne Dis..

[bib79] Wiesinger A., Wenderlein J., Ulrich S., Hiereth S., Chitimia-Dobler L., Straubinger R.K. (2023). Revealing the tick microbiome: Insights into midgut and salivary gland microbiota of female *Ixodes ricinus* ticks. Int. J. Mol. Sci..

[bib80] Zając Z., Obregon D., Foucault-Simonin A., Wu-Chuang A., Moutailler S., Galon C. (2023). Disparate dynamics of pathogen prevalence in *Ixodes ricinus* and *Dermacentor reticulatus* ticks occurring sympatrically in diverse habitats. Sci. Rep..

